# Metabolic Programming of Macrophages: Implications in the Pathogenesis of Granulomatous Disease

**DOI:** 10.3389/fimmu.2019.02265

**Published:** 2019-10-04

**Authors:** Jayne Louise Wilson, Hannah Katharina Mayr, Thomas Weichhart

**Affiliations:** Center for Pathobiochemistry and Genetics, Institute of Medical Genetics, Medical University of Vienna, Vienna, Austria

**Keywords:** macrophage, immunometabolism, granuloma, tuberculosis, schistosomiasis, sarcoidosis

## Abstract

Metabolic reprogramming is rapidly gaining appreciation in the etiology of immune cell dysfunction in a variety of diseases. Tuberculosis, schistosomiasis, and sarcoidosis represent an important class of diseases characterized by the formation of granulomas, where macrophages are causatively implicated in disease pathogenesis. Recent studies support the incidence of macrophage metabolic reprogramming in granulomas of both infectious and non-infectious origin. These publications identify the mechanistic target of rapamycin (mTOR), as well as the major regulators of lipid metabolism and cellular energy balance, peroxisome proliferator receptor gamma (PPAR-γ) and adenosine monophosphate-activated protein kinase (AMPK), respectively, as key players in the pathological progression of granulomas. In this review, we present a comprehensive breakdown of emerging research on the link between macrophage cell metabolism and granulomas of different etiology, and how parallels can be drawn between different forms of granulomatous disease. In particular, we discuss the role of PPAR-γ signaling and lipid metabolism, which are currently the best-represented metabolic pathways in this context, and we highlight dysregulated lipid metabolism as a common denominator in granulomatous disease progression. This review therefore aims to highlight metabolic mechanisms of granuloma immune cell fate and open up research questions for the identification of potential therapeutic targets in the future.

## Introduction

At its core, a granuloma is a compact and organized structure formed by the initial aggregation of macrophages in response to a persistent stimulus (1, 2). A multitude of stimuli have been reported to evoke a granulomatous reaction, including infectious agents, the best studied of which are *Mycobacterium tuberculosis* and the parasitic trematode genus *Schistosoma*, non-infectious foreign bodies such as silica, pollutants, dust and biomedical implants (3, 4), as well as autoimmune or inflammatory diseases of unknown etiology, including sarcoidosis and Crohn's disease. It has also been suggested, particularly in the case of sarcoidosis, that a combination of infectious and non-infectious factors could be responsible for disease pathogenesis, with evidence suggesting *Propionibacterium acnes* or mycobacterial infection as potential environmental triggers (5).

Depending on the nature and persistence of the inciting stimulus, the macrophages at the core of the granuloma can undergo a series of distinct morphological changes, the most prominent involving epithelioid cell differentiation; an enigmatic process for which the exact trigger and molecular mechanisms have yet to be elucidated. The resultant macrophages, termed “epithelioid,” are characterized by a hypertrophic, flattened appearance, diffuse cytoplasm and elongated nuclei, as well as epithelial-like interdigitated cell membranes, enabling the cells to interlace and form an epithelioid barrier to wall off the persistent antigen (1, 6, 7). These epithelioid macrophages can further develop into multinucleated giant cells via as yet undefined mechanisms; however, cell-cell fusion (8, 9) and cytokinesis failure (10) have been proposed. Furthermore, in the case of tuberculosis granulomas, macrophages can transform into foam cells as a result of their enhanced accumulation of lipids as the infection progresses (11).

As the granuloma matures, a number of additional cells can be recruited to the structure—a process that depends heavily on the nature of the inciting agent. Such cells include granulocytes, monocytes, dendritic cells, B and T cells, NK cells and fibroblasts (1, 12, 13), which surround the macrophage core resulting in a complex and highly organized structure. A subsequent and final stage of granuloma maturation involves the development of pathological structures as a result of dysregulated inflammation, which are associated with tissue damage and morbidity. One such feature is the formation of a fibrotic outer capsule that occurs in granulomas of diverse etiology, including sarcoidosis and schistosomiasis. Another pathological feature, which develops primarily in granulomas of infectious origin, is the formation of necrotic regions within the central core of the granuloma. This is a characteristic that is well-studied in the case of tuberculosis, during which necrosis is associated with a failure of the immune response and results in bacterial dissemination and patient morbidity (14).

Due to the major clinical role of granulomas in an array of disease pathologies, the morphological properties of granuloma formation have been extensively studied. However, the fundamental and molecular core mechanisms involved in the granulomatous immune response are only starting to emerge. Our group recently identified the mechanistic target of rapamycin (mTOR) complex 1 (mTORC1) signaling pathway as a molecular mechanism in the initiation and maintenance of granulomas in mice, as well as its activation in sarcoidosis patients suffering from a progressive form of the disease (15). mTOR is well-known to sense and integrate a range of environmental signals to regulate cellular metabolism and cell growth in many cell types (16, 17). However, our work specifically revealed an involvement of macrophage mTORC1 signaling in the context of granuloma formation and development (15), thus highlighting the potential importance of macrophage immunometabolic responses in granulomatous disease. While mTORC1 was also recently identified as a driver of foam cell biogenesis in tuberculosis (18), the relevance of mTOR signaling in schistosomiasis remains to be elucidated. Likewise, adenosine monophosphate-activated protein kinase (AMPK) has been reported to orchestrate lipid catabolism and oxidative phosphorylation (OXHPOS) in tuberculosis (19, 20); however, an involvement of this metabolic regulator in schistosomiasis and sarcoidosis has barely been defined. Interestingly, peroxisome proliferator receptor gamma (PPAR-γ)-signaling has been implicated in the progression of all three diseases. As PPAR-γ is a master regulator of lipid homeostasis (21), this strongly suggests an involvement of lipid metabolism in the pathogenesis of granulomas.

Indeed, the significance of macrophage metabolic plasticity dependent on disease pathology is now widely accepted and the field of immunometabolism has gained momentum in recent years. New findings have emerged that shed light on the complex molecular mechanisms underpinning macrophage function across numerous diseases states; however, at present, such responses are exquisitely linked to the current M1/M2 framework of macrophage polarization. In this review, we focus on the current literature describing the immunometabolic programming of macrophages—as the workhorses at the forefront of granuloma formation, development and maintenance (1, 13)—in the best characterized examples of granulomatous disease: tuberculosis, schistosomiasis and sarcoidosis. In particular, we highlight literature pertaining to the role of PPAR-γ and lipid metabolism in the pathogenesis of these diseases, which suggests dysregulated lipid metabolism as a key contributor to granuloma fate.

## A Prelude to M1/M2 Polarization and Its Immunometabolic Nature

At present, the concept of M1/M2 macrophage polarization, albeit oversimplified and based primarily on *in vitro* data, still remains one of the best means by which macrophage activation can be described. Classically activated M1 macrophages function at the crux of host defense by eliciting essential pro-inflammatory responses and bridging innate and adaptive immunity. In contrast, alternatively activated M2 macrophages are crucial to immune regulation by promoting the resolution of inflammation and preventing an exacerbated, chronic inflammatory state, as well as the maintenance of tissue homeostasis by inducing tissue healing and remodeling (22–24). M1 and M2 polarization states are additionally defined by distinct metabolic profiles that are crucial in driving the differential activation of macrophages (25). It is important to note, however, that while the scheme of M1/M2 polarization has been very useful in furthering our understanding of macrophage function and biology, *in vivo* and human studies point toward a larger, less distinct spectrum of polarization states (26, 27). Accordingly, it is becoming more common to refer to the subcategories, which include M1a, M1b, and M2a-c that are, in part, defined by their differential expression of chemokine receptors (28). Transcriptomic analyses have revealed that there are six main polarization states in humans that are highly sensitive to certain stimuli, and thus the nomenclature has also been adapted to “M(LPS),” “M(IL-4)” and others (29). In addition, there is significant evidence to suggest that these polarization states have a high plasticity and do not represent a terminal differentiation (30).

Nevertheless, the pathological consequences of the immunometabolic nature of macrophage polarization in granulomas are only just starting to emerge, with the tubercle granuloma currently constituting the best-studied example. Thus, while the metabolic landscape of granuloma macrophages in a human disease scenario will be much more complex, the simplified M1/M2 dogma and the use of *in vivo* models currently comprise the primary framework for discussing macrophage metabolism in the context of granulomatous disease. Below, we therefore provide a brief overview of the key immunometabolic features of M1/M2 macrophage polarization as an introduction to the subsequent discussion on their contributing roles in the pathology of tuberculosis, schistosomiasis and sarcoidosis.

### Classically Activated (M1) Macrophages

M1 macrophage polarization is typically stimulated by inflammatory cytokines including interferon-gamma (IFN-γ) and/or tumor necrosis factor (TNF) in combination with Toll-like receptor (TLR) ligation (31). This leads to NF-κB signaling via the phosphorylation of Inhibitor of (I)κB kinase, and the activation of several interferon-regulatory factors (IRFs) (32), which are signature genes of the M1 macrophage phenotype. Although both TLR ligation and IFN-γ signaling can individually and redundantly induce the phenotype (31), it was recently shown in tumor-associated macrophages that both mechanisms may need to work synergistically to produce an effective, highly pro-inflammatory M1 switch (33). Metabolically, M1 macrophage activation classically involves the Warburg effect. This metabolic switch is well-known in oncology and refers to a distinct metabolic profile in tumor cells undergoing uncontrolled division, during which cells utilize glycolysis for rapid ATP generation in the presence of oxygen, termed “aerobic glycolysis” (34). In macrophages, this switch drives the essential increase in ATP production required to support the innate immune response to infectious insult and/or tissue injury, supporting the rapid production of inflammatory cytokines and enhancing phagocytosis (25). Both TLR ligation and LPS-induced increased hypoxia-inducible factor 1-α (HIF-1α) expression are associated with the Warburg effect in macrophages (35, 36). Specifically, HIF-1α links the effector functions of M1 macrophages to their metabolic profile by both activating glycolytic enzymes and directly promoting transcription of the inflammatory mediator IL-1β (36). Moreover, when HIF-1α is overexpressed in macrophages, mitochondrial OXPHOS is suppressed and the cells enter a highly glycolytic and inflammatory state with a clear M1 phenotype (37). It must be noted, however, that the Warburg effect has almost exclusively been observed *in vitro* and in cancer cells, and it has been proposed that M1 macrophages only exhibit a “Warburg-like” phenotype (38).

### Alternatively Activated (M2) Macrophages

M2 macrophage polarization is brought about by the cytokines IL-4 and IL-13 released from CD4^+^ Th2 cells (39, 40). The IL-4/13 receptor activates insulin receptor substrate 2 (IRS2), leading to the upregulation of key M2 markers: Arg-1, RELMα, and Ym1. The IL-4/13 receptors signal via signal transducer and activator of transcription 6 (STAT6) to activate phospholipases and GATA binding protein 3 (GATA3), which are required for the production of chemokines and the anti-inflammatory cytokine IL-10 (41, 42). There is also evidence that IL-10 can produce an M2 or M2-like phenotype via the phosphorylation of STAT3, leading to the production of IL-10 in an autocrine manner (43, 44). Unlike their M1 counterparts, M2 macrophages are defined by their intact TCA cycle, which allows for the generation of FADH2 and NADH and the production of high ATP yields through OXPHOS. Concomitantly, M2 macrophages display increased glycolysis as a carbon source (15, 45). Importantly, an intact TCA cycle allows for the glycosylation of M2-specific mannose and lectin receptors by UDP-GlcNAc (46). OXPHOS in M2 macrophages is supported by fatty acid oxidation (FAO), and M2 polarization is marked by increased expression of the lipid scavenger receptor CD36, as well as a dependence on cell-intrinsic lysosomal lipolysis (38, 45, 47). M2 macrophages are also known to consume more glutamine than other macrophage phenotypes (48). Glutamine augments the production of UDP-GlcNAc (46) and glutaminolysis has been shown to produce α-ketoglutarate, a major metabolite and component of the TCA cycle (49). Glutamine-derived α-ketoglutarate promotes specific histone demethylation and transcription of M2 genes (49, 50).

## Tuberculosis and the Immunometabolic Response: A Balancing Act

Tuberculosis is an infectious disease caused by the bacillus *Mycobacterium tuberculosis* (Mtb) and is one of the most frequent etiological triggers of granuloma formation. It is the leading cause of mortality from a single infectious agent and was responsible for an estimated 1.7 million deaths worldwide in 2017 (51). However, only a small proportion (5–10%) of the infected population will develop active tuberculosis, with a greater incidence in individuals infected with HIV or suffering from malnutrition or diabetes (51). It has therefore long been established that the outcome of Mtb infection depends upon a complex and dynamic interplay between host and pathogen (52–54), with the degree of bacterial virulence and host resistance defining the pathogenesis of disease (55–57).

### Initial Host Responses to Mtb Infection

Tuberculosis is typically a disease of the lungs initiated upon inhalation of airborne droplets containing the tubercle bacilli at an estimated infectious dose of just a single bacterium (58, 59). The bacteria are subsequently deposited in the airways where they are phagocytosed by resident alveolar macrophages and transported into the lung parenchyma (60, 61). The generation of TNF-α and inflammatory chemokines by the infected macrophages then drives a localized inflammatory response involving the recruitment of additional mononuclear cells (52), including interstitial macrophages, monocytes, neutrophils, and dendritic cells, many of which Mtb subsequently infects (54). Dendritic cells transfer Mtb from the lungs to the local draining lymph nodes (62) for subsequent priming of naive T cells and the adaptive immune response (63), the development of which is unusually delayed during Mtb infection, taking up to 5–6 weeks in humans (64, 65). The latter has been attributed to successful immune evasion by the bacteria, which is thought to seek refuge within lung phagocytes (54). Thus, although Mtb is known to infect multiple immune cells in the lung, macrophages are considered to be the key players in the balance between bacterial containment and disease progression (66). Moreover, increasing evidence from non-human primate and/or human patient granulomas suggests that mycobacterial burden, as well as advancement to active disease, is associated with an increased proportion of alternatively activated M2 to classically activated M1 macrophages (67–69). We will explore this further in the following subsections, with a particular focus on the emerging importance of the immunometabolic characteristics of M1- vs. M2-like macrophages on the outcome of mycobacterial infection.

### Distinct Immunometabolic Responses of Pulmonary Macrophages to Mtb

Tissue resident alveolar macrophages are the first cells to encounter invading mycobacteria after aerosol infection (54). Several studies in diverse model systems, encompassing mycobacterial infection of human alveolar macrophages (70–72), mouse models (73, 74) and zebrafish larvae (75), demonstrate an initial mycobactericidal role that is conserved in multiple tissue resident macrophages across species. However, these reports contend with numerous contradictory studies that render the alveolar macrophage as a favorable niche for the growth and survival of mycobacteria, acting as a shield against the bactericidal activity of subsequently recruited immune cells, as well as activation of the Th1 response (54).

At steady state, alveolar macrophages do not conform to the classical definition of M1 or M2 polarization, but instead exhibit a hybrid phenotype that affords a degree plasticity in keeping with their critical role in maintaining lung homeostasis (76, 77). It is logical that as sentinels of the lung, alveolar macrophages would mount an initial pro-inflammatory response to invading pathogens. Taking into account their potential to switch between activation states, it may not be surprising if they then re-polarize to a more M2-like status in response to a persistent pathogen to protect against hyperinflammation and exacerbated lung immunopathology. Such a switch toward an M2 state is particularly pertinent in the case of Mtb infection, considering the ability of mycobacteria to manipulate host immune responses to favor their own survival (52). Recent data suggesting alveolar macrophages are more permissive to Mtb replication than recruited monocyte-derived interstitial macrophages (78) highlight distinct metabolic programming as a critical factor in the differential antimycobacterial capacity of these two macrophage populations. This study utilized transcriptomic and subsequent pathway enrichment analyses, as well as functional *in vivo* and *in vitro* experiments employing inhibitors of different metabolic pathways, to reveal the metabolic preferences of Mtb-infected alveolar vs. interstitial macrophages. This work demonstrated that Mtb-infected alveolar macrophages preferentially utilize FAO and exhibit enhanced fatty acid uptake, as well as higher bacterial replication rates, than interstitial macrophages. As it is well-known that Mtb accesses host-derived fatty acids and cholesterol as a primary carbon source (79–82), the fact that alveolar macrophages exhibit enhanced fatty acid uptake may explain why they are more permissive to Mtb replication. Furthermore, Huang et al. (78) showed that a FAO inhibitor, etomoxir, almost entirely abolished the production of IFN-β in Mtb-infected BMDMs. As type I interferon responses have been shown in multiple studies to be detrimental during tuberculosis [reviewed by (83)], this may explain how FAO can also contribute to Mtb survival. Conversely, interstitial macrophages were primarily glycolytic, which was shown to restrict mycobacterial growth (78), presumably due to the pro-inflammatory cytokine signals associated with glycolysis as well as the comparatively lower uptake of fatty acids by these cells. This is in agreement with several studies that demonstrate the requirement of glycolysis for Mtb growth control (84–86). Huang et al. (78) also showed that depletion of alveolar macrophages in Mtb-infected mice reduced bacterial burden, whereas depletion of the interstitial macrophage population was detrimental, resulting in enhanced bacterial survival. Thus, in this setting, alveolar macrophages appear to function as M2-like nutritionally permissive hosts in which mycobacteria can evade pro-inflammatory action and replicate relatively unperturbed, which is in contrast to the more growth-restrictive environment within pro-inflammatory interstitial macrophages. These data are supported by additional studies of alveolar or M2 macrophage depletion in animal models of pulmonary tuberculosis, which was associated with an enhanced Th1 response, reduced bacterial burden in the lung and protection against tuberculosis-induced lethality (87, 88). Granuloma formation was also found to be defective in mice deficient in alveolar macrophages, while the attraction and activation of T cells in the lung, as well as the numbers of polymorphonuclear cells, were enhanced (87). This is in contrast to the depletion of activated M1-like macrophages, which was detrimental and resulted in enhanced bacterial survival (88).

Such immunometabolic divergence of the two pulmonary macrophage populations in response to Mtb infection suggests that the developmental origin of granuloma macrophages plays a role in disease progression. Indeed, the role of ontogeny in macrophage functionality and metabolism during infectious insult has been discussed in a recent review (89). Furthermore, it should be noted that while the Warburg effect is considered to be primarily an *in vitro* phenomenon, the *in vivo* relevance of this bioenergetic phenotype is starting to emerge in the literature. Specifically, during the establishment of chronic Mtb infection in mice (up to 30 days after low-dose aerosol infection), transcriptomic and histological analysis of Mtb-infected lung tissue revealed a HIF-1α-dependent enhancement of glucose uptake and glycolysis, as well as lactate formation and export (84). This was coupled with a concomitant down-regulation of pyruvate dehydrogenase complex (PDC), TCA cycle enzymes and OXPHOS. Moreover, defects in macrophage glycolytic capacity have been associated with the enhanced susceptibility of cigarette smokers to Mtb infection (86). In this study, extracellular flux analysis of Mtb-infected human alveolar macrophages isolated from the bronchoalveolar lavage (BAL) fluid of smokers and non-smokers revealed an impairment of metabolic activity in the alveolar macrophages of the smokers, including reduced glycolytic response and spare respiratory capacity, which was accompanied by a weakened inflammatory response.

### Biphasic Immunometabolic Response of Individual Mtb-Infected Macrophages

Further to the immunometabolic distinction between pulmonary macrophages of different lineages, increasing evidence points toward a time-dependent blend of M1 and M2 responses to Mtb infection within individual granuloma macrophages (90–92), which may further explain the paradox of alveolar macrophage functionality during infection. One such study utilized high-throughput capped analysis of gene expression (deepCAGE) technology to investigate the promoter-based transcriptional landscape of Mtb-infected macrophages (91). This work revealed drastic gene expression alterations that included up-regulation of genes involved in M1-related immune response and inflammation, as well as M2-related cell wounding and apoptosis. Furthermore, to delineate macrophage responses to Mtb infection, authors of a recent review (92) comprehensively analyzed the metabolic patterns reported in transcriptome databases and supplementary data files from studies of primary macrophage Mtb infection in the literature. The authors report on a biphasic response marked by a defensive M1 phenotype during the early phase of *in vitro* infection (up to 8 h post-infection), followed by a switch to an M2-driven adaptation/resolution phase as the infection progresses (24–48 h after infection). The early pro-inflammatory phase was characterized by upregulation of genes indicating a classical Warburg shift in metabolism, including *Hif1*α, as well as genes encoding glucose uptake transporters (GLUT1 and GLUT6), hexokinases (HK1 and HK2), phosphofructokinase liver (PFKL; from the phosphofructokinase-1 (PFK-1) family), 6-phosphofructo-2-kinase/fructose-2,6 biphosphatase 3 (PFKFB3; an essential enzyme from the PFK-2 family that is responsible for elevated glycolytic flux), and the major lactate secretion transporter member 4 (MCT4). Consistent with the profile of M1 macrophage polarization, this up-regulation of Warburg-associated genes was coupled with the downregulation of genes encoding mitochondrial enzymes and proteins, including the PDC, TCA cycle enzymes (aconitase 2 [ACO2], isocitrate dehydrogenase 2 [IDH2] and subunits of the succinate dehydrogenase [SDH] complex), as well as multiple components of respiratory chain complexes.

These findings are consistent with the current knowledge of the molecular mechanisms underpinning the metabolic switch in M1 macrophages, which are brought about by two distinct “breakpoints” in the TCA cycle (25). The first breakpoint occurs at the conversion of citrate to α-ketoglutarate, which is catalyzed by isocitrate dehydrogenase 1 (IDH1). IDH1 is downregulated 7-fold in M1 macrophages compared to non-polarized macrophages, while the ratio of citrate to α-ketoglutarate is tripled (46). Itaconate, generated from the citrate accumulated as a result of this metabolic break (93), is a powerful bactericidal agent produced by M1 macrophages that functions as a potent inhibitor of bacterial isocitrate lyase (ICL). ICL, an enzyme that facilitates retention of carbon from fatty acids via the glyoxylate shunt (94), has been implicated in the control of Mtb infection (95, 96) due to its requirement for fatty acid catabolism by the bacteria (96). In this study, Muñoz-Elías and McKinney show that deletion of the genes that encode two ICL isoforms in Mtb (*icl1* and *icl2*) reduced growth and survival of the bacteria in murine macrophages and in human monocyte-derived macrophages, as well as the bacterial load in mouse lungs.

The second breakpoint in the TCA cycle occurs at the succinate-fumarate conversion step, which is catalyzed by SDH, as demonstrated by the accumulation of mitochondrial succinate in LPS-stimulated macrophages (36). Succinate accumulation occurs partially as a result of the aforementioned citrate-induced generation of itaconate, which downregulates SDH directly (97). Succinate can also be produced directly from glutamine in a TCA cycle-independent, γ-aminobutyric acid (GABA)-dependent manner via the GABA shunt (36, 46, 49, 50). Increasing evidence supports the role of succinate as an important metabolic signal linking metabolism and immunity (92, 98), as succinate has been shown to stabilize HIF-1α and its proinflammatory effects, in particular its induction of IL-1β expression (99, 100). In addition to the identification of genes involved in these classical pathways, Shi et al. (92) report the modulation of a diverse array of genes associated with a number of pro-inflammatory processes affected as a result of these TCA cycle breaks. Such processes include an augmented oxidative stress response coupled with an increase in antioxidant defense, as well as the synthesis of pro-inflammatory bioactive lipids (including long chain fatty acyl-CoAs, phospholipids and prostaglandins), and arginine uptake and metabolism.

Consistent with the metabolic profile of alternative macrophage activation, the adaptation/resolution phase transition in Mtb-infected macrophages was marked by gene expression changes signifying a reduction in glucose uptake and dampened glycolytic metabolism, with a concomitant recovery of the TCA cycle and OXPHOS (92). The latter coincided with induction of *Pgc1b* (92), which encodes PPAR-γ coactivator-1β (PGC-1β), a key player in mitochondrial biogenesis and oxidative metabolism that has been implicated in alternative macrophage activation (47). The PPAR-γ coactivator family of transcription factors (PGC-1) are potent anti-inflammatory regulators and enhancers of OXPHOS in various cell types, including macrophages, where retroviral transfection with PGC-1β drives non-polarized cells toward an anti-inflammatory M2 phenotype characterized by enhanced OXPHOS and arginase-I expression (47).

Studies focusing on the behavior of Mtb during its infection cycle are also suggestive of a biphasic immunometabolic response of individual macrophages to Mtb infection. Following macrophage invasion, Mtb undergoes a series of physiological adaptations that accompany distinct phases of its infection process, from initial infection to intra-macrophage adaptation and eventual establishment of successful, productive disease. Transcriptional profiling of Mtb, in conjunction with the use of a clock plasmid to measure bacterial replication and death rates, revealed a clear distinction in bacterial survival and response to host phagocyte function in a 2-week *in vitro* infection model encompassing this multi-phasic infection process (101). For the first 2 days post-infection of primary BMDMs, the bacteria encountered a “bottleneck” during which Mtb killing outweighed its relatively high replication rate (101); a finding that is emulated *in vivo* by rapid bacterial growth and pronounced bacterial killing in the mouse lung during the first 2 weeks of infection (102). Reduced survival of Mtb during early *in vitro* infection was attributed to Mtb stress due to the initial macrophage arsenal, inferred by up-regulation of Mtb genes involved in general stress response, carbon metabolism, oxidative stress, iron storage, as well as hypoxia and nitrosative stress (101). These findings are in accordance with an earlier transcriptomic dataset that was also acquired 2-day after Mtb infection of murine BMDMs (103).

The bottleneck observed by Rohde et al. (101) was followed by a period of reduced replication but enhanced bacterial survival (intracellular adaptation phase), which progressed over time to a period of extended growth resulting in established macrophage infection. Interestingly, only a subset of Mtb genes were upregulated early (by day 2 post-infection) and remained elevated over the remaining 2-week infection period, including genes involved in fatty acid and cholesterol metabolism, secreted antigens and regulators. Furthermore, many of the general stress response genes observed during early infection were markedly down-regulated during the adaptation and establishment phases. Such modifications of the Mtb response over time are consistent with an M1-M2 switch in macrophage activation, and suggest that Mtb exploits the proposed biphasic response of host macrophages in order to establish productive infection.

### Foamy Macrophages and Lipid Metabolism in Tuberculosis

A characteristic feature of tuberculosis pathogenesis is the formation of foam cells (lipid-laden macrophages) that are associated with tubercle granuloma necrosis (11) due to their contribution to caseum formation, which is defined as an accumulation of necrotic debris at the core of the granuloma. The latter promotes inflammation, tissue injury, and the eventual cavitation of the granulomatous structure, resulting in transmission of live bacilli and tuberculosis disease [reviewed by (104)]. Although the role of foamy macrophages in the progression of tuberculosis is clear, the mechanisms controlling foam cell biogenesis remain ill-defined for this disease. However, a recent study using multiple Mtb-infection models has shed light on these mechanisms, as well as the lipid composition of tuberculosis foam cells (18). This work demonstrated that foamy macrophages in necrotizing granulomas in tuberculosis lung lesions accumulate triglycerides (TAG), and exhibit a multispecies TAG profile that is conserved in rabbits, non-human primates and humans. Elevated levels of TAG were also detected in Mtb-infected human monocyte-derived macrophages, and were accompanied by higher lipid content and enhanced expression of TAG biosynthesis genes. Mechanistically, this TAG accumulation was mediated by TNFR signaling via downstream activation of the caspase cascade and mTORC1. Inhibition of these pathways was shown to significantly reduce lipid content in these cells, and up-regulation of the pathways was also observed in transcriptomic data from human tuberculosis lung tissue (18). Interestingly, an involvement of mTORC1 was previously implied by histological analysis of a human tuberculosis lung sample, which revealed mTORC1 activation in foamy macrophages (105). Furthermore, this key metabolic sensor has been shown to promote lipogenesis, in particular TAG biosynthesis, in obesity, diabetes, cancer and neurodegenerative disorders, while blocking lipolysis and β-oxidation (106). Indeed, Mtb and other mycobacterial species induce mTOR in macrophages (107, 108), and rapamycin inhibition of mTOR has been shown to decrease mycobacterial viability (107).

It is also interesting to note that mycobacterial infection of macrophages induces the expression and activation of PPAR-γ (109), a hallmark of alternative macrophage activation and a master regulator of lipid metabolism that controls fatty acid uptake, storage and lipogenesis (21). PPAR-γ has been implicated in mycobacterial disease progression by modulating host cell metabolism toward lipid droplet formation, as well as diminishing the pro-inflammatory immune response to favor bacterial survival (109–113). Moreover, a link between mycobacterial virulence and host PPAR-γ expression and activation during infection has been proposed. For instance, the virulent H37Rv strain of Mtb was shown to induce PPAR-γ expression, and attenuated growth of virulent Mtb was observed in human macrophages following PPAR-γ deletion (111), as well as in the lungs of macrophage-specific PPAR-γ-deficient mice (113). In contrast, attenuated *M. bovis* bacillus Calmette-Guerin (BCG) up-regulated PPAR-γ to a lesser extent (111) and *M. smegmatis*, an avirulent mycobacterial strain, failed to induce PPAR-γ expression (110, 111). Taken together, these findings suggest that PPAR-γ may also contribute to foam cell biogenesis in tuberculosis granulomas. This may additionally explain the link between PPAR-γ and tuberculosis pathogenesis, which is suggested by the correlation between Mtb virulence and PPAR-γ activity. Furthermore, enhanced expression of PPAR-γ has been reported in adipogenesis, and this was shown to be mTOR-dependent (114, 115), which raises the question as to whether PPAR-γ plays a role in mTORC1-mediated foam cell formation in tuberculosis. Interestingly, PPAR-α, one of the three PPAR isoforms alongside PPAR-γ and PPAR-β and a key driver of fatty acid β-oxidation (116), was recently reported to play an essential role in host innate immune defense against Mtb and BCG (117). In this study, the PPAR-α-mediated antimycobacterial response of BMDMs was attributed to enhanced autophagy, lysosomal biogenesis and phagosome maturation, as well as suppression of exacerbated inflammation via activation of transcription factor EB (TFEB). Moreover, PPAR-α promoted lipid catabolism, mitochondrial respiration and fatty acid β-oxidation in mycobacteria-infected macrophages (117). Thus, it appears that PPAR-γ and PPAR-α play opposing roles during mycobacterial infection, which is due, in part, to their distinct effects on host lipid homeostasis.

AMPK is another key metabolic regulator that has gained traction in tuberculosis research, although the literature on its role in other granulomatous diseases is sparse. AMPK is an important coordinator of M2 polarization and lipid catabolism, and its effects can overlap, enhance or antagonize those of mTOR and the PPAR family [reviewed by (118)]. During Mtb infection, AMPK activation increases OXPHOS, FAO and also antimicrobial autophagy by interacting with PGC-1α, as well as inhibiting Mtb-induced mTOR activation (19, 20). It is well-established that autophagy constitutes a successful antimycobacterial host response (119), therefore it is no surprise that Mtb has developed mechanisms to protect itself against this process. One such mechanism is the induction of microRNA expression in macrophages that directly silence AMPK (20). A second mechanism could be the activation of mTOR (19, 120), which is a well-known inhibitor of autophagy (121, 122). Indeed, mTORC1 has been reported to inhibit PPAR-α (123), which may constitute a mechanism by which mTORC1 inhibits autophagy in Mtb-infected cells. In comparison, mTORC1 is known to enhance the expression of PPAR-γ (114, 115), which supports an interplay between mTORC1 and PPAR-γ in the formation of foamy macrophages during Mtb infection.

**Figure 1:**
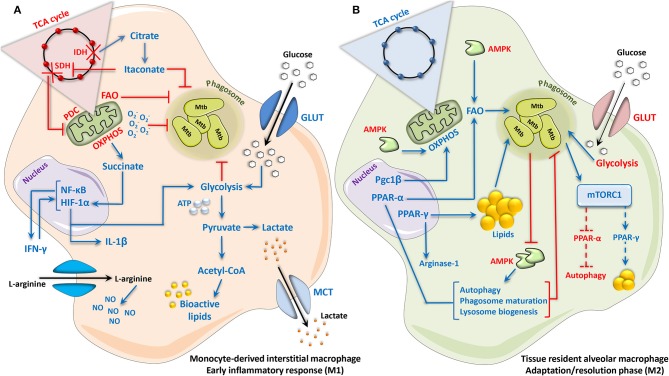
*Mycobacterium tuberculosis* elicits a biphasic immunometabolic response in host macrophages. **(A)** Monocyte-derived interstitial macrophages, and macrophages during the acute phase response to Mtb infection, adopt an M1-like phenotype characterized by a Warburg-like switch in metabolism. Enhanced HIF-1α-mediated glycolytic activity and glucose uptake results in the rapid generation of ATP to support the pro-inflammatory reaction, which occurs simultaneous to the increased generation and export of lactate. Concomitant reductions in OXPHOS, PDC, and FAO occur as result of two consecutive breaks in the TCA cycle at the conversion of citrate to α-ketoglutarate (α-KG) and succinate to fumarate, as well as the down-regulation of the TCA cycle enzymes involved in these reactions: IDH and SDH, respectively. Accumulation of the TCA intermediate citrate as a result of the first breakpoint in the cycle (citrate to α-KG) leads to the generation of itaconate, which inhibits SDH directly as well as Mtb survival by blocking bacterial isocitrate lyase and consequently fatty acid catabolism by the bacteria. Accumulation of succinate as a result of the second break in the TCA cycle (succinate to fumarate), as well as direct inhibition of SDH by itaconate, stabilizes HIF-1α and its pro-inflammatory activity. The uptake and metabolism of arginine are additionally increased, promoting the production of NO via iNOS/NOS2. Together, these events culminate in the generation of a potent antimycobacterial response marked by the generation of pro-inflammatory cytokines, ROS/RNS and bioactive lipids, which in parallel with increased glycolysis and reduced FAO, result in Mtb growth control. **(B)** Tissue-resident alveolar macrophages, and macrophages entering an adaptation/resolution phase during chronic Mtb infection, adopt an M2-like phenotype characterized by an intact TCA cycle and enhanced FAO-driven OXPHOS, with a concomitant reduction in glycolytic activity and glucose uptake. This coincides with increased expression and activity of Pgc1β, which promotes mitochondrial biogenesis and oxidative metabolism. During the infection process, Mtb augments PPAR-γ expression, resulting in a weakened inflammatory response, as well the formation and accumulation of lipid droplets within the cell, thus providing a favorable niche for the growth and survival of Mtb. PPAR-α can also be activated in macrophages during Mtb infection, which enhances autophagic, lysosomal and phagosomal processes that contribute to Mtb growth control. Likewise, cytosolic AMPK, while enhancing OXPHOS and FAO, also promotes antimycobacterial autophagy, and is therefore directly inhibited by Mtb. mTORC1, induced by Mtb, supports bacterial survival by promoting lipogenesis and blocking autophagy, potentially due to its interactions with PPAR-γ and PPAR-α, respectively. Dotted lines represent interactions inferred from the literature. Up- and downregulation are indicated by blue and red, respectively. Acetyl-CoA, acetyl coenzyme A; AMPK, adenosine monophosphate activated protein kinase; ATP, adenosine triphosphate; FAO, fatty acid oxidation; GLUT, glucose transporter; HIF-1α, hypoxia-inducible factor 1-alpha; IDH, isocitrate dehydrogenase; IFN-γ, interferon gamma; IL, interleukin; iNOS, inducible nitric oxide synthase; MCT, monocarboxylate transporter; Mtb, *Mycobacterium tuberculosis*; mTORC1, mammalian/mechanistic target of rapamycin complex 1; NF-κB, nuclear factor ‘kappa-light-chain-enhancer’ of activated B-cells; NO, nitric oxide; NOS2, NO synthase 2; OXPHOS, oxidative phosphorylation; PDC, pyruvate dehydrogenase complex; Pgc1β, peroxisome proliferator-activated receptor gamma coactivator 1-beta; PPAR, peroxisome proliferator-activated receptor; ROS, reactive oxygen species; RNS, reactive nitrogen species; SDH, succinate dehydrogenase; TCA, tricarboxylic acid.

These findings highlight not only the importance of macrophage lipid metabolism in the pathogenesis of tuberculosis, but also the critical involvement of mTORC1 and PPAR-γ in the survival and maintenance of Mtb during disease progression. The additional identification of AMPK and PPAR- α as host protective metabolic signaling pathways further accentuates the important link between immunometabolic signaling and the outcome of granulomatous disease. An overview of the Mtb-macrophage interactions described in this section is provided in Figure 1.

## Schistosomiasis: A Story of Eggs and Fats

The second major cause of infectious granulomas, schistosomiasis (or bilharzia), is an endemic tropical disease of significant morbidity, mortality and socioeconomic impact. Like other helminth-borne infections, schistosomiasis occurs mainly in the southern hemisphere, where it is considered endemic in over 50 countries and affects over 200 million people, making it the most significant parasitic infection after malaria (124). Schistosomiasis is transmitted to humans via the aquatic larvae of several geographically distinct species of the *Schistosoma* trematodes. The most prominent causative agents of human schistosomiasis are *S. mansoni, S. japonicum*, and *S. haematobium*, all of which employ freshwater snails as intermediate hosts. Upon subcutaneous infection with the swimming larval stage, known as cercariae, the worms mature in the lung over the course of a month. During this time, the infection elicits an acute type-1 inflammatory response, which, in the case of *S. mansoni* and *S. japonicum*, subsides when the adult schistosomes travel to the hepatic and mesenteric vasculature to mate and lay eggs (125). *S. haematobium* instead colonizes the urogenital tract. Egg deposition into perivascular tissues is accompanied by an acute type-2 response about 2 months post-infection, which finally becomes a lower-grade chronic disease marked by chronic pain, anemia, diarrhea, hepatomegaly and malnutrition (126). During this type-2 antihelminthic response, schistosome egg antigens induce rapid periovular granuloma formation that encases the eggs. Periovular granulomas protect the host from continuous exposure to toxic egg antigens and reduce immunopathology, but hepatic granulomas frequently become fibrotic if untreated, leading to portal hypertension and eventually liver cirrhosis with significant morbidity (127). Schistosome granulomas therefore have advantages and disadvantages for the host and their usefulness as a containment strategy has been discussed at length (128–130).

### Cellular Composition of Schistosome Granulomas

Typically, periovular granulomas consist of alternatively activated M2 macrophages, Th2 CD4^+^ T cells and eosinophils (128). Although M2 macrophages are considered the key component of schistosome granulomas (131), granuloma composition is variable, with hepatic granulomas showing a higher cell heterogeneity and intestinal granulomas containing largely macrophages (132). Based on inverse proportions of eosinophils, macrophage numbers also appear to be higher in the granulomas of naturally-infected wild water rats (*Nectomys squamipes*) than in chronic granulomas of experimentally-infected mice (133), indicating that there may be significant differences between experimental and natural granuloma formation that must be taken into account. Schistosome egg-derived antigens such as omega-1 play a major role in driving a highly Th2-skewed antihelminthic immune response at 5–6 weeks after the initial infection, causing local CD4^+^ T helper cells to release IL-4 and IL-13 (134, 135). At the same time, intercellular adhesion molecule 1 (ICAM-1) is upregulated on sinusoidal endothelial cells surrounding newly deposited eggs (136). This milieu of cytokines and chemokines results in the T cell and STAT6-dependent recruitment of Ly6C^hi^ monocytes to the periovular space, where they differentiate into granuloma macrophages with only a minor contribution from resident hepatic macrophages (131, 137–139). In the case of *S. japonicum* infection, parasite antigens can also signal via TLR2 to promote M2 polarization (140). Contrastingly, Tundup et al. (141) showed that the TLR co-receptor CD14 is highly upregulated in hepatic macrophages upon *S. mansoni* infection and acts as a crucial negative regulator of M2 polarization, possibly as part of a parasitic defense mechanism against granuloma formation (141). CD14, in turn, induces uptake of oxidized low-density lipoproteins (oxLDL), cholesterol and lipids into macrophages (142).

### Lipid Metabolism and PPAR-γ-Signaling in Schistosomiasis

*S. mansoni* infection was recently linked to specific metabolic changes in hepatic macrophages, where granuloma formation around schistosome eggs was associated with differential lipid and cholesterol metabolism (143). This is supported by multiple studies that have highlighted the significance of PPAR-γ in the context of schistosomiasis. PPAR-γ is a hallmark of M2 polarization and globally regulates lipid uptake and metabolism (144, 145). It is activated by a multitude of immunometabolic ligands, including various unsaturated fatty acids, lipoproteins, eicosanoids, flavonoids and the amino acids glutamine and arginine (146). Although the precise role of PPAR-γ in macrophages depends on their lineage, polarization state and post-translational modifications (145, 147), PPAR-γ is generally considered to be an anti-inflammatory receptor known to limit M1 activation, for instance by transrepression and ubiquitination of NF-κB (145, 148). Importantly, lipids secreted from the tegument of *S. mansoni* adults were shown to directly activate PPAR-γ in macrophages, leading to upregulation of arginase-1 expression and other M2 markers *in vitro* (149). This study did not clarify whether this effect is only achieved in the presence of adult worms, or whether similar PPAR-γ-activating lipids are also found on the eggs. However, both live and inactivated *S. mansoni* eggs have been reported to induce a 7-fold increase in PPAR-γ expression in human liver cell cultures (150). PPAR-γ activation is a classical M2 macrophage marker and M2 macrophages are known to preferentially utilize FAO. While mice with a macrophage-specific PPAR-γ knockout show a downregulation of genes for both synthesis and oxidation of fatty acids in non-infectious settings (144), *S. mansoni*-infected human liver biopsies revealed a specific downregulation of FAO-related genes, including acetyl-CoA acyltransferase 2 (ACAA2) and acyl-CoA synthetase long-chain family member 1 (ACSL1) (151). Additionally, a secretory protein of *S. japonicum* inhibited PPAR-α, a key promoter of FAO, in a colitis mouse model (152), indicating that downregulation of FAO may be directed by the parasite itself. These findings support the notion that macrophage polarization during schistosomiasis is complex, with a potential decrease in FAO despite many classical M2 signatures (153).

**Figure 2:**
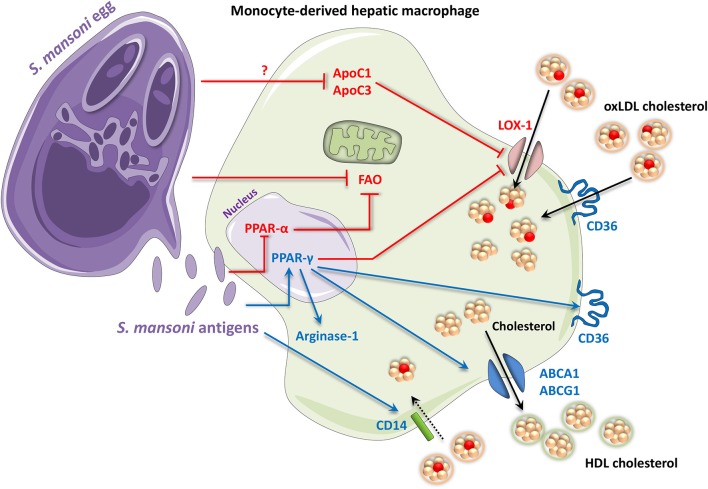
Periovular granuloma macrophages in schistosomiasis assume an M2-like phenotype with altered lipid metabolism. Antigens from *Schistosoma mansoni* eggs (and adults) activate PPAR-γ signaling. Some *Schistosoma* products have also been shown to inhibit signaling by the FAO inducer, PPAR-α. PPAR-γ contributes to an M2 phenotype by upregulating M2 markers including arginase-1. It also promotes expression of the scavenger receptor CD36, which takes up free fatty acids and oxidized LDL cholesterol. In contrast, PPAR-γ inhibits oxLDL cholesterol uptake via the LOX-1 receptor. *S. mansoni* itself also attenuates LOX-1 by decreasing ApoC1 and ApoC3 via an unknown mechanism. Additionally, *S. mansoni* infection also leads to an upregulation of CD14, which has in turn been shown to promote uptake of oxLDL and other lipids in macrophages. HDL cholesterol efflux may also be increased through the PPAR-γ-mediated upregulation of ABCA1 and ABCG1, contributing to an anti-atherogenic phenotype. Up- and downregulation are indicated by blue and red, respectively. ABC, adenosine triphosphate-binding cassette transporter; Apo, apolipoprotein; CD, cluster of differentiation; FAO, fatty acid oxidation; HDL, high-density lipoprotein; LOX-1, lectin-like oxidized low-density lipoprotein receptor 1; oxLDL, oxidized low-density lipoprotein; PPAR, peroxisome proliferator-activated receptor.

It is now well-established that infection with *S. mansoni* alters host lipid metabolism globally by reducing total cholesterol, low-density lipoprotein (LDL) and triglycerides in the plasma of humans and apolipoprotein E (apoE)-deficient or high-fat diet-fed mice (154–156). In particular, PPAR-γ activation during schistosomiasis has been shown to counteract atherosclerosis and other high-fat diet-induced pathologies (157, 158). PPAR-γ has been strongly linked to these conditions, and mice with macrophage-specific PPAR-γ knockouts become susceptible to diet-induced obesity and insulin resistance (144). Peritoneal macrophages with a conditional PPAR-γ knockout have markedly reduced expression of cholesterol transport genes, such as the ATP-binding cassette transporters (ABC) A1 and G1, as well as reduced cholesterol efflux (157, 159, 160). Similar effects were shown in hepatic macrophages during schistosomiasis, where a recent study reports downregulation of a number of genes involved in cholesterol metabolism, including *APOC1* and *APOC3* (143), which are known contributors to inflammatory atherosclerosis (161, 162). A recent RNA interference study revealed that ApoC1 in particular is a key promoter of oxLDL cholesterol uptake into macrophages via the lectin-like oxLDL receptor-1 (LOX-1), which is in turn inhibited by PPAR-γ (163, 164). Thus, *S. mansoni* infection blocks macrophage oxLDL cholesterol uptake by inhibiting ApoC1 and ApoC3 and may at the same time facilitate cholesterol efflux via ABCA1 and ABCG1. This suggests that unlike in tuberculosis, schistosomiasis macrophages do not accumulate lipids (see Figure 2). In accordance with this, there are no reports to date of *S. mansoni* or *S. japonicum* leading to foam cell formation. The rarer *S. mekongi*, which does not promote fibrosis, appears to be the only member of its genus to induce foam cells, although this phenomenon has not yet been investigated further (165).

Mechanistically, *S. mansoni* infection leads to significant upregulation of CD14, which induces macrophage uptake of oxLDL cholesterol and lipids (141, 142). Additionally, PPAR-γ is known to mediate transcription of the *Cd36* scavenger receptor gene in macrophages (166), which promotes uptake of oxLDL cholesterol and provides a stepping stone for the development of atherosclerosis (158). A PPAR-γ response element in the *Cd36* gene means that PPAR-γ directly promotes CD36 transcription following PPAR-γ activation by oxLDL, constituting a positive feedback loop for cholesterol accumulation (158, 167, 168). In mice infected with *S. mansoni* while on an atherogenic high-fat diet, significant lipid uptake was found to be induced in the outermost cell layers of hepatic periovular granulomas; however, the plasma ratio of HDL to LDL was still improved (156). Unfortunately, this study did not determine which cells were responsible for the increased lipid uptake. However, as the experiments were performed only 7 weeks after *S. mansoni* infection, when granulomas are still somewhat immature and not yet associated with additional leukocytes (128), it is likely that the responsible cells were either eosinophils or macrophages. Another study showed that *S. mansoni* infection can induce lipid uptake and retention in hepatic stellate cells (150). Therefore, the lipid droplet-positive cells identified by Stanley et al. (156) could be non-granulomatous hepatic cells, although this would not account for their circular distribution around the granulomas. Since lipid uptake was only induced in the outermost cell layers of granulomas (156), there may be an as-of-yet unknown significance of physical proximity between the egg and the surrounding macrophages. While it is known that female schistosomes take up large amounts of fatty acids in order to produce eggs, it is not established whether eggs, which themselves contain large amounts of fatty acids, could extract and ingest lipids from surrounding macrophages (169, 170). It may be the case that CD36-induced oxLDL uptake, while pro-atherogenic in arterial walls, allows some sequestration of oxLDL cholesterol in non-migratory macrophages, thus contributing to the anti-atherogenic effect of schistosomiasis (171).

Overall, *S. mansoni* is characterized by modifications in the PPAR-γ network of lipid metabolism, during which oxLDL cholesterol uptake via ApoC1 and ApoC3 is directly suppressed, while uptake via CD14 and CD36 may be enhanced due to PPAR-γ signaling. Since PPAR-γ can directly activate multiple HDL cholesterol efflux channels, and there appears to be limited lipid accumulation and no evidence of foam cell formation in schistosome macrophages, this suggests that there may be a significant efflux of cholesterol from granuloma macrophages during schistosomiasis. The expulsion of HDL cholesterol could explain the anti-diabetic and anti-atherogenic effects observed in response to PPAR-γ agonists such as rosiglitazone (172–174).

### PPAR-γ in *Schistosoma*-Induced Fibrosis

Macrophage lipid metabolism may also be involved in the formation of fibrosis, the major pathology of chronic schistosomiasis, as suggested by several studies using pharmacological PPAR-γ induction. For instance, in a mouse model of *S. japonicum*-induced liver fibrosis, the PPAR-γ agonist rosiglitazone was able to reduce liver fibrosis and extracellular matrix (ECM) deposition, which was attributed to decreased inflammatory signaling (175). Although this study did not link these effects to granuloma macrophages, but rather to hepatic stellate cells and myofibroblasts, ECM deposition and local synthesis have been described as key components in both sarcoid and infectious lung granulomas (176). Similar to the effects of rosiglitazone, the PPAR-γ agonist pioglitazone reduced hepatic and splenic histopathology in a mouse model of *S. japonicum* infection by increasing the proportion of regulatory T cells and inducing the polarization of mannose receptor-positive M2 macrophages (177). Another PPAR-γ agonist, telmisartan, reduced both hepatic fibrosis and granuloma diameter in *S. mansoni*-infected mice, although it did not synergistically increase the efficacy of praziquantel treatment, an antihelminthic drug commonly used to treat schistosomiasis (178). These findings may seem contradictory, seeing as PPAR-γ is known to induce M2 polarization, which is necessary for host survival during initial infection (179, 180), and also contributes to fibrosis by promoting fibroblast hyperactivity through TGF-β signaling (181, 182). At the same time, pharmacological activation of PPAR-γ has been shown multiple times to decrease histopathology and fibrosis in schistosomiasis and some renal pathologies (183). Where PPAR-γ agonists have failed to ameliorate fibrosis, this has been speculatively attributed to the simultaneous activation of pro-fibrotic M2 macrophages (184, 185). It is therefore likely that this dual role of PPAR-γ is due to its activity in various cell types. For instance, activation and overexpression of PPAR-γ has been shown to act directly on both human and mouse fibroblasts to decrease fibrosis by reducing growth factor expression, mitosis, collagen secretion, and responsiveness to TGF-β (186, 187).

While these studies do not explicitly link the anti-fibrotic effects of different PPAR-γ agonists to immune cell lipid metabolism, it is clear that PPAR-γ plays a significant role in schistosomiasis pathogenesis and future work investigating how macrophage lipid metabolism affects fibrosis should prove interesting. Taken together, it is becoming apparent that *Schistosoma* infection has highly complex consequences for lipid metabolism, at both the cellular and global level (summarized in Figure 2), and more work is required to elucidate the exact metabolic pathways that characterize schistosome granulomas.

## Sarcoidosis: A Disease Lacking an Immunometabolic Switch?

Unlike tuberculosis and schistosomiasis, which are triggered by known pathogens and are typically organ-specific, sarcoidosis is an enigmatic multi-systemic disease of unknown origin. It is characterized by the development and accumulation of epithelioid, non-caseating (non-necrotic) granulomas typically found in the lungs; however, sarcoid granulomas can present almost anywhere in the body, with other notable sites including the eyes, skin and lymph nodes (188). The disease is increasingly difficult to treat once it develops from a self-limiting to progressive state, with pulmonary and cardiac involvement representing the most frequent causes of patient morbidity and mortality (189). Due to the unknown etiology of sarcoidosis, there is currently no therapeutic approach targeting the pathogenetic mechanisms (190). However, the occurrence of familial forms of the disease (191, 192) suggests that a genetic background may play a pathological role, and pine pollen (193), microbial infection (specifically *P. acnes* and mycobacteria) (5) as well as air pollutants (comprising mineral, micro or nanoparticles) (3) are increasingly regarded as strong environmental trigger candidates (188, 194, 195). Pollution, in particular, gained momentum among clinicians and biologists when a recrudescence of sarcoidosis was observed in the aftermath of the World Trade Center tragedy in subjects exposed to particulate matter (3). Although the exact relative contributions of genetic and environmental factors are unknown, it has been suggested that genetic factors account for 66% of disease susceptibility in monozygotic twins (191).

### Immunological Profile of Sarcoidosis

At the cellular level, sarcoid granulomas are characterized by a strong Th1/Th17 phenotype (196). Once again, this sets sarcoidosis apart from tuberculosis and schistosomiasis, which are both marked by an acute Th1 and M1 phase that later progresses to a predominantly M2 phenotype. It has long been known that the pro-inflammatory milieu of IL-12, IL-17, TNFα, and IFNγ upregulate adhesion molecules on macrophages to promote aggregation, cell-to-cell contact and fusion (197, 198). In 2011, however, macrophages and multinucleated giant cells from granulomas of patients with systemic neuromuscular sarcoidosis were shown for the first time to be M2-polarized with high CCL18 expression and resistance to conversion by Th1 cytokines (199). More recently, immunohistochemical analysis also revealed M2 macrophage activation in lung and lymph node samples from pulmonary sarcoidosis patients (200). Advanced sequencing techniques confirmed that a CD163-positive macrophage fraction of peripheral blood mononuclear cells (PBMCs), isolated from sarcoidosis patients and treated with purified protein derivative (PPD), exhibits a partial M2 profile with upregulation of IL-13 downstream pathways (201, 202). Furthermore, the pro-inflammatory cytokine IL-17, well-established in sarcoidosis pathology, has also been shown to induce an M2-like phenotype in macrophages (203, 204). It is important to note that sarcoidosis is frequently linked to clinical anergy, which presents as the loss of skin test reactivity to PPD and other antigens in sarcoidosis patients. While CD4^+^ T cells and dendritic cells have been implicated in this anergic response [reviewed by (205)], the findings described above suggest that anti-inflammatory macrophages may also contribute to the paradoxical diminished immunity often observed in sarcoidosis patients.

### Dysregulation of Lipid Metabolism in Sarcoidosis

The M2 profile of macrophages reported for some sarcoid granulomas may also be associated with the expression and activity of PPAR-γ, as PPAR-γ deficiency was observed in alveolar macrophages of pulmonary sarcoidosis patients (206) and its expression was shown to negatively correlate with disease severity (207). Furthermore, polymorphisms in the *Pparg* gene and the gene encoding its transcriptional coactivator PPAR-γ coactivator 1-α (*Ppargc1a*) were identified at a higher frequency in sarcoidosis patients compared with healthy subjects (208). Thus, a genetic defect in PPAR-γ signaling may be a predisposing factor for the development of severe sarcoidosis. Additional work utilizing diverse mouse models supports the role of PPAR-γ in lung immunopathology (157, 159, 209–212). In one such study employing a multiwall carbon nanoparticle (MWCNT) model, which recapitulates the chronicity of human granulomas (211), PPAR-γ expression and activity was significantly reduced in alveolar macrophages following oropharyngeal instillation of MWCNT in wild type animals. Accordingly, increased pulmonary granuloma formation, as well as expression of pro-inflammatory markers in granulomatous lung tissue and BAL, were observed in macrophage-specific PPAR-γ-deficient mice (termed *PPAR-*γ^fl/fl^, *Lyz2*-Cre) (153). Moreover, increased numbers of Th1 lymphocytes were observed in BAL fluid extracted from *PPAR-*γ^fl/fl^, *Lyz2*-Cre mice compared to wild type controls. PPAR-γ deficient BAL cells also displayed elevated expression of inducible nitric oxide synthase (iNOS) and IFN-γ, as well as Th1-associated cytokines (209). Reconstitution of PPAR-γ by lentiviral transduction in the same study significantly reduced the expression of pro-inflammatory mediators and decreased the number of BAL lymphocytes by 90%. Taken together, these data suggest an important role of PPAR-γ in the regulation of pulmonary inflammation and maintenance of lung homeostasis.

As mentioned earlier, chronic inflammation typically triggers a heightened activation state in fibroblasts, lending to substantial deposition of ECM components at the site of injury and consequent development of fibrosis (213, 214). Thus, PPAR-γ deficiency in sarcoid granulomas of patients presenting severe forms of the disease may play a role in the characteristic Th1/M1 bias, and consequently the development of fibrosis that is typically associated with disease pathogenesis. This notion correlates with the observation that NF-κB activation is increased in BAL samples from sarcoidosis patients (215). As described previously in this review, transrepression of NF-κB by PPAR-γ is well-established, thus increased and/or uncontrolled activation of NF-κB as a result of PPAR-γ deficiency could be one mechanism by which continuous inflammation occurs. Indeed, in a study coupling a computational model of granuloma formation and function, termed *GranSim* (216), with macrophage polarization data from non-human primate tuberculosis granulomas, continuous or increased NF-κB signaling was shown to exacerbate inflammation, resulting in uncontrolled bacterial growth and dissemination (68).

Mechanistically, PPAR-γ maintains pulmonary lipid homeostasis via alveolar macrophage liver X receptor-alpha (LXR-α) and ABCG1 (217), which, alongside ABCA1, is critical for macrophage efflux of cholesterol and phospholipids (218). Alveolar macrophages from sarcoidosis patients and mice following MWCNT instillation show diminished expression of ABCG1 and ABCA1, and the deficiency of these transporters in MWCNT-instilled mice correlates with increased alveolar macrophage lipid accumulation (210). In accordance with these findings, activation of the PPARγ-ABCG1 pathway by the PPARγ agonist rosiglitazone tempers MWCNT-induced granulomatous inflammation by significantly attenuating alveolar macrophage activation, pulmonary granuloma formation and pulmonary lipid dysregulation (212). Thus, the consequence of PPAR-γ deficiency in sarcoid granuloma macrophages appears to be manifold, resulting in both an uncontrolled inflammatory response and dysregulation of macrophage lipid metabolism.

Indeed, a number of proteins involved in lipid metabolism have been identified as contributing factors in the pathogenesis of sarcoidosis, including apoA1, fatty acid-binding protein 4/perilipin 2, 8-isoprostane, zinc-α2 glycoprotein and serum amyloid A (SAA) (219). In particular, SAA, a highly inducible acute-phase reactant and amyloid precursor protein (220), is a well-known modulator of the innate immune response, inflammation and apolipoprotein metabolism (221), and it has been suggested as a potential biomarker for sarcoidosis due to its significantly higher serum concentrations in patients vs. healthy subjects (222). However, SAA is not a diagnostic gold standard as it is also elevated in a number of other inflammatory conditions, namely arthritis and systemic lupus erythematosus (223). In tissue samples from sarcoidosis patients, SAA was shown to localize to macrophages and giant cells within granulomas, but it also correlated with the number of CD3^+^ cells and a local Th1 response (224). In this study, SAA was shown to promote chronic inflammation in sarcoid granulomas via TLR2 signaling and activation of NF-κB, as well as cytokine production (224). Contrastingly, recent evidence suggests that during inflammation, SAA acts synergistically with secretory phospholipase-A to remove cell membrane debris, which suggests a partial involvement in anti-inflammatory repair processes (225). Interestingly, SAA has also been suggested to be responsible for the low HDL cholesterol and apoA1 levels observed in patients with active sarcoidosis (226, 227), which has been linked to an increased risk of atherosclerosis in sarcoidosis patients (219). This is in contrast to schistosomiasis, which has been reported to counteract atherosclerosis by altering global host lipid metabolism and reducing serum cholesterol levels (154).

### Metabolic Signaling as a Driver of Sarcoidosis Disease Progression

As mentioned previously in this review, Linke et al. (15) identified an involvement of macrophage-specific mTORC1 signaling in the initiation and maintenance of granulomas. In this study, constitutive activation of mTORC1 in murine macrophages was achieved via myeloid-specific deletion of the gene encoding its upstream inhibitor tuberous sclerosis complex 2 (Tsc2) (termed *Tsc2*^fl/fl^, *Lyz2*-Cre mice). This genotype induced hypertrophy and enhanced cell proliferation in macrophages while reducing their apoptotic capacity. As a result, the *Tsc2*^fl/fl^, *Lyz2*-Cre mice exhibited spontaneous formation of non-caseating (non-necrotic), epithelioid granulomas that were observed in multiple organs. In the lung specifically, the granuloma macrophages were shown to consist of M2-like alveolar macrophages. Thus, the disease pathology seen in the *Tsc2*^fl/fl^*,Lyz2*-Cre mice strongly resembles the histological phenotype of sarcoidosis. Mechanistically, genes involved in both glycolysis and OXPHOS were enriched in transcriptomic datasets from *Tsc2*^fl/fl^*,Lyz2*-Cre BMDMs compared with *Tsc2* floxed control (termed *Tsc2*^fl/fl^) BMDMs, and the enhancement of both metabolic pathways was confirmed by extracellular flux analysis. Furthermore, the uptake of glucose was higher in *Tsc2*^fl/fl^*,Lyz2*-Cre BMDMs and absolute glucose levels were decreased in both Tsc2-deficient macrophages as well as in the lungs of Tsc2-deficient animals, while mitochondrial mass and mitochondrial spare respiratory capacity were elevated (15). These findings are in accordance with the identification of increased levels of pyruvate in sarcoidosis patient sera, which is indicative of enhanced glycolytic activity (228). This study also reported an increase in serum metabolites that indicate enhanced mitochondrial FAO, as well as a decrease in serum levels of the key TCA cycle intermediate succinate (228). RNA-Seq data from sarcoidosis patient monocytes further revealed a dysregulation of OXPHOS and FAO pathways (229). Interestingly, the metabolic alterations described by Linke et al. were shown to be CDK4-dependent and crucial for the enhanced proliferation and reduced apoptosis of Tsc2-deficient macrophages. This study additionally identified mTORC1 activation, macrophage proliferation and glycolysis as hallmarks of disease progression in human sarcoidosis patients (15). Clinical involvement of mTORC1 has since been substantiated by its recent identification in RNA-Seq gene set enrichment data from cutaneous sarcoidosis patients (230), as well as whole exome sequencing and pathogenicity network analysis of familial cases of sarcoidosis (231). It should also be noted that successful treatment of a sarcoidosis patient with the mTOR inhibitor rapamycin has previously been reported (232). However, the molecular mechanisms underpinning mTOR involvement in human sarcoidosis remain to be elucidated.

Among other pathways, the involvement of mTORC1 points to a role of glycolytic metabolism in sarcoidosis pathogenesis. Importantly, the PI3K/Akt/mTOR axis, and mTORC1 specifically, have been shown to promote the production of the key glycolytic regulator HIF-1α irrespective of oxygen concentrations (233–235). In accordance with this, a recent study demonstrated elevated protein levels of HIF-1α, as well as HIF-1α signaling pathway components such as HIF-1β, HIF-2α, and p300, in alveolar macrophages from sarcoidosis patients cultured under normoxic conditions (236). HIF-1α protein expression was further confirmed in granulomatous lung tissue from sarcoidosis patients, and was found to be localized to alveolar macrophages and multinucleated giant cells (236). As described previously, HIF-1α promotes pro-inflammatory macrophage functions by activating glycolytic enzymes and stimulating the production of IL-1β (36). Indeed, Talreja et al. (236) demonstrated that in alveolar macrophages from sarcoidosis patients, increased levels of HIF-1α correlated with augmented Glut1 protein expression and elevated levels of IL-1β. Furthermore, siRNA inhibition of HIF-1α in patient PBMCs significantly diminished the production of IL-1β, IL-17 and IL-6 (236). Thus, HIF-1α signaling appears to play a critical role in the pro-inflammatory milieu that defines sarcoidosis pathology, and provides an explanation for the enhanced glycolysis reported in the aforementioned studies.

These findings highlight the complexity of the metabolic landscape of granuloma macrophages both *in vivo* and in human disease. In particular, they emphasize the oversimplification of Warburg metabolism in M1 macrophages, which is classically defined by a simultaneous increase in glycolysis and decrease in OXPHOS. This accentuates the importance of future work that examines more closely the complexity of macrophage metabolism *in vivo* and in the context of individual human diseases. This is, however, in contrast to Mtb infection, during which the Warburg-like immune response has been documented and supported by *in vivo* data (84). However, this difference may simply be due to the infectious nature of Mtb and a typical pro-inflammatory reaction to initial infection, which is absent in sarcoidosis. It is also important to note that some of the sarcoidosis studies mentioned in this section utilized either patient PBMCs or patient sera, and therefore describe the peripheral/global metabolic alterations associated with sarcoidosis. The role of each of the identified metabolic pathways within the sarcoid granuloma environment, and their contribution to sarcoid granuloma macrophage function specifically, still has to be assessed. Nevertheless, the observations that glycolysis, OXPHOS and FAO may all occur simultaneously during sarcoidosis disease progression, and that these pathways are associated with both pro- and anti-inflammatory functions, may somewhat explain the paradoxical immunoregulation and anergy that are frequently associated with sarcoidosis.

**Figure 3:**
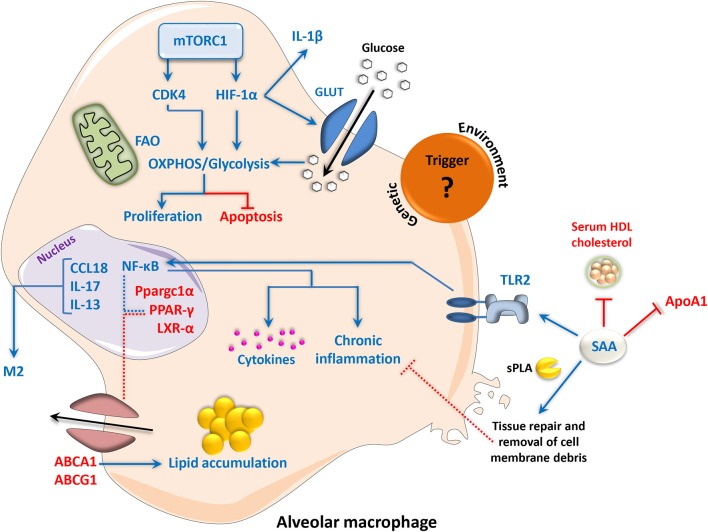
Metabolic signaling and chronic inflammation in sarcoidosis disease progression. The etiological trigger of sarcoidosis remains unknown; however, environmental and genetic factors have been proposed. Deficiency of PPAR-γ and its transcriptional coactivator Ppargc1α in alveolar macrophages has been implicated in disease severity, resulting in the alleviation of NF-κB transrepression and enhancement of a pro-inflammatory phenotype. Reduced expression of the cholesterol and lipid transporters ABCG1 and ABCA1 and disruption of LXR-α signaling, which are involved in the maintenance of lipid homeostasis by PPAR-γ, leads to lipid accumulation in macrophages. NF-κB can also be activated via TLR2 signaling in response to increased levels of SAA, leading to the generation of pro-inflammatory molecules. SAA can reduce serum HDL cholesterol and ApoA1, increasing the risk of atherosclerosis in sarcoidosis patients. However, SAA can also work with sPLA to promote the enzymatic digestion and removal of cell debris, which contributes to the anti-inflammatory processes of wound healing and tissue repair. In sarcoid-like granuloma macrophages, mTORC1 promotes cell proliferation and inhibits apoptosis via CDK4-dependent enhancement of glycolysis and OXPHOS. mTORC1 also directly promotes the function of HIF-1α, which in turn contributes to glycolysis and inflammation. An M2-like phenotype in sarcoid granulomas has additionally been reported, involving CCL18, IL-13, and IL-17. Up- and downregulation are indicated by blue and red, respectively. Dotted lines represent interactions inferred from the literature. ABC, adenosine triphosphate-binding cassette transporter; Apo, apolipoprotein; CCL18, chemokine (C-C motif) ligand 18; CDK, cyclin-dependent kinase; HDL, high-density lipoprotein; HIF-1α, hypoxia-inducible factor 1-alpha; IL, interleukin; GLUT, glucose transporter; LXR, liver X receptor; mTORC1, mammalian/mechanistic target of rapamycin complex 1; NF-κB, nuclear factor ‘kappa-light-chain-enhancer’ of activated B-cells; OXPHOS, oxidative phosphorylation; PPAR, peroxisome proliferator-activated receptor; SAA, serum amyloid A; sPLA, secretory phospholipase A; TLR, toll-like receptor.

An overview of the findings described in this section is provided in Figure 3.

## Common and Distinct Metabolic Features of Tuberculosis, Schistosomiasis, and Sarcoidosis

PPAR-γ signaling and dysregulated lipid metabolism are the overriding common features of each disease discussed in this review. However, these pathways are distinctly modulated depending on the disease, as well as disease severity. Such differences may be due to the granuloma-inciting agent and, in particular, the ability of mycobacteria and *Schistosoma* species to manipulate host macrophage responses to promote their own survival. For instance, both tuberculosis and schistosomiasis are defined by an acute inflammatory phase followed by a pathogen-induced shift toward M2-like macrophage polarization, marked by the activation of PPAR-γ. In tuberculosis, PPAR-γ activation appears to be virulence-dependent, leading to lipid droplet accumulation and consequent foam cell formation that are associated with the development of necrosis, which is characteristic of tuberculosis pathogenesis. However, in schistosomiasis there appears to be only minor CD14/CD36-driven lipid accumulation that is confined to certain areas of the granuloma, with a potential efflux of cholesterol from macrophages and no reports of foam cell formation or a correlation with pathogenicity for the major *Schistosoma* species. This is further compounded by the up-regulation of FAO in Mtb-infected macrophages compared to its down-regulation in *Schistosoma*-infected livers, which may, in part, constitute a protective mechanism in tuberculosis foamy macrophages to counteract some of the lipid accumulation that occurs during infection. In contrast to tuberculosis and schistosomiasis, sarcoidosis exhibits defective or deficient PPAR-γ signaling that is associated with disease severity. However, this is not surprising considering the critical role of PPAR-γ in the M1-M2 metabolic switch in macrophages, and the chronic inflammatory nature of sarcoid granulomas in comparison to tuberculosis and schistosomiasis. Furthermore, the expression of SAA, which contributes to chronic inflammation, is greater in sarcoidosis than in other granulomatous diseases (224). SAA has also been associated with the increased prevalence of atherosclerosis in sarcoidosis patients by lowering serum levels of HDL and apoA1. This is again in contrast to schistosomiasis, which has been shown to reduce atherogenesis potentially due to the expulsion of HDL cholesterol from schistosome granuloma macrophages. This may be linked to enhanced PPAR-γ expression during *Schistosoma* infection that will, in turn, lead to increased expression of the cholesterol transporters ABCA1 and ABCG1, thus enabling HDL cholesterol efflux. Whereas, down-regulation of PPAR-γ, and accordingly ABCA1 and ABCG1, in macrophages from sarcoidosis patients and mice imply a decrease in the efflux of HDL cholesterol from these macrophages. Interestingly, FAO is also up-regulated in sarcoidosis (228), which may again constitute a homeostatic response of sarcoid granuloma macrophages to the increased lipid accumulation that occurs due to PPAR-γ deficiency. These findings highlight the importance of PPAR-γ in the control of lipid homeostasis, and how its dysregulation in a disease scenario can have immunometabolic consequences that directly impact macrophage effector functions and disease outcome.

In further support of the involvement of macrophage metabolic signaling in granulomatous disease progression, macrophage mTORC1 has recently been implicated in tuberculosis and sarcoidosis pathogenesis. However, the metabolic consequences of mTORC1 activation in each case appears to be distinct. During Mtb infection, this key metabolic sensor plays a role in foamy macrophage formation, whereas in sarcoidosis mTORC1 promotes OXPHOS and glycolysis. As mTOR is a key intracellular nutrient sensor, these differences suggest that macrophages in tubercle and sarcoid granulomas may have a distinct nutritional content, which may again be due to the ability of Mtb to manipulate host cells to promote its own survival. On that note, Mtb-induced dysregulation of autophagy in macrophages is an established mechanism by which Mtb evades the host immune response. Interestingly, a link between mTOR and autophagy was recently proposed in familial cases of sarcoidosis (231). Therefore, the role of mTOR in these diseases may in fact be manifold, and the connection between mTOR and autophagy in both tuberculosis and sarcoidosis suggests that this could be a key molecular pathway in the pathogenesis of granulomatous diseases. In schistosomiasis, however, very little is known about the function of mTOR. In dendritic cells, which play a major role in Th2 priming, mTOR inhibition by both rapamycin and torin-1 has been shown to increase IL-4 expression (237). However, while schistosome infection leads to an M2/Th2 profile shift in dendritic cells, the parasite antigens appear to do so in an mTOR-independent manner (237). One study reports that 90 % of mTOR pathway genes were downregulated in liver biopsies from human schistosomiasis *japonica* patients (151), even though *in vitro* work suggests that IL-13 induced by parasitic infections may enhance the expression of the mTORC2 protein Rictor (238). mTOR expression is also enhanced in *S. haematobium*-associated bladder cancer, although this may be a general feature of malignancy rather than schistosomiasis (239). Recently, mTOR signaling in myeloid cells has also emerged as an attractive target for the treatment of hepatic fibrosis (240, 241).

## Future Perspectives

While research on the immunometabolic features of the pathogenesis of granulomatous diseases is developing, particularly in the case of tuberculosis, much more work is required to further our understanding of the exact roles of the different metabolic pathways in each disease. As this review highlights, the metabolic plasticity of macrophages allows for distinct metabolic phenotypes of individual granulomas that are highly dependent on the inciting agent. Identifying the key common and distinct features will not only be critical in defining potential therapeutic targets in the future, but may also provide an insight into how we could control the more enigmatic forms of granulomatous disease, such as sarcoidosis and Crohn's disease, for which the etiological triggers remain elusive. In tuberculosis and schistosomiasis, for example, the up-regulation of PPAR-γ results in a controlled immune response that prevents against exacerbated tissue damage by inducing an M2-like macrophage polarization state. While this role of PPAR-γ can be detrimental for pathogen clearance by the host in the case of infectious granulomas, it points toward a potential mechanism by which the chronic inflammation in sarcoidosis could be alleviated. Evidence for a role of the other PPAR isoforms (PPAR-α and PPAR-β) in granulomas of schistosomiasis and sarcoidosis is sparse in the literature and needs to be assessed in more detail. This is especially relevant considering that in tuberculosis the observed antimycobacterial function of PPAR-α is in direct contrast to the implicated function of PPAR-γ in mycobacterial disease progression, as well as the role of PPAR-α in promoting autophagy in Mtb-infected cells. While the specific significance of PPAR-α has not yet been studied in schistosomiasis, Sj16, an *S. japonicum* secretory protein, has been shown to inhibit PPAR-α (152). This lends weight to the notion that PPAR-α may play a role in schistosomiasis, and one that may also contrast with PPAR-γ. Future research should aim to examine the precise role(s) of the different PPARs in granulomatous disease, which may further our understanding of the complex lipid metabolism that is observed in each of the diseases discussed in this review. Because of the emerging significance of PPAR-γ in particular, it is also important to reiterate its well-established link with mTOR [e.g., (242, 243)]. This connection suggests that research directed at determining the precise role of mTOR in schistosomiasis should prove fruitful in the future and could highlight pathways of potential significance to tuberculosis and sarcoidosis.

AMPK could, unfortunately, not be assessed for a comparison between the granulomatous diseases discussed in this review, as it has been described almost exclusively for tuberculosis. However, while there is no research to date on the role of AMPK in sarcoidosis, IL-7-induced AMPK signaling in schistosomiasis *japonicum* has been shown to counteract macrophage autophagy and potentiate liver damage (244). This is in direct contrast with the multiple reports of AMPK promoting macrophage autophagy during mycobacterial infection (20). Nevertheless, AMPK could prove a valuable component of granuloma immunometabolism as it has been linked to both PPAR-γ (245) and mTOR (118, 246). In cancer cell lines, for instance, AMPK and AMPK activators such as metformin have an inhibitory effect on PPAR-γ activity (247). In rat liver samples, AMPK activation was shown not only to suppress PPAR-γ activity, but also to decrease fatty acid synthesis while enhancing the beta-oxidation enzyme carnitine palmitoyltransferase (248). While it remains to be seen whether these findings are translatable into granuloma research, AMPK should prove an interesting target for future research.

The comparison between schistosomiasis and tuberculosis highlights an additional key difference relating to the structure of their respective granulomas and the ontogeny of the participating macrophages, which are likely influenced by the respective pathogens. For instance, tuberculous granulomas differ greatly based on the stage of disease, whereas the composition of schistosome granulomas depends on where the eggs are deposited. Additionally, in tuberculosis, granulomas appear to involve more tissue-resident pulmonary macrophages, while schistosome granulomas consist almost exclusively of monocyte-derived macrophages. Therefore, it will be interesting to investigate the activation and metabolic programming of macrophages dependent on the local environment of the granuloma vs. macrophage ontogeny. Metabolic differences have also been shown within the layers of individual granulomas, so the metabolic profiles of macrophages and/or the additional immune cells of the granuloma dependent on their spatial orientation within the granulomatous structure will also require characterization. This can also be applied to the different pathological structures of granulomas, particularly fibrotic, foam cell-containing and necrotic granulomas. Deciphering the metabolic pathways underlying the formation of these distinct structures will allow us to determine why some granulomas are more effective than others, and perhaps how to redirect granulomas toward a less harmful phenotype.

Finally, it is important to note that there is a significant epidemiological overlap between tuberculosis, HIV, malaria and schistosomiasis (249). It would therefore be interesting for future research to consider a schistosomaiasis-tuberculosis co-infection approach. Even in urban settings of the southern hemisphere, studies report that over 60% of Mtb-exposed children also suffer from a helminth infection, the vast majority of which are caused by *Schistosoma* species (250). Furthermore, it was recently shown that infection with *S. haematobium* induces long-term, persistent epigenetic alterations resulting in a weakened inflammatory response and increased susceptibility to tuberculosis (251). Hence, research on tuberculosis therapies and vaccine candidates has made a point to assess the effect of *Schistosoma* species on tuberculosis disease progression (252). As both pathogens form granulomas but affect macrophage metabolism in a vastly different manner, it would be interesting to analyze granuloma formation in co-infection settings, particularly with regard to lipid metabolism.

Overall, the findings highlighted in this review demonstrate the importance and complexity of immunometabolic signaling in granulomatous diseases of different etiology. This emphasizes the need for future research that deciphers more closely the contribution of different metabolic pathways to each individual disease, as we all as the regulation of these pathways by PPARs, mTOR, and AMPK. Furthermore, it is paramount that the role of key metabolic pathways and regulators are then verified *in vivo*, and where possible, by human studies. Building comprehensive metabolic networks that define granuloma macrophage function will enable us to identify novel therapeutic targets in the future.

## Author Contributions

JW conceived of, and with HM wrote, reviewed and edited the manuscript. TW reviewed and edited the manuscript.

### Conflict of Interest

The authors declare that the research was conducted in the absence of any commercial or financial relationships that could be construed as a potential conflict of interest.
